# Risk factors for short birth interval: A hospital-based cross-sectional study among women in the Nkongsamba Health District, Littoral Region, Cameroon

**DOI:** 10.1371/journal.pgph.0001446

**Published:** 2023-07-31

**Authors:** Atem Bethel Ajong, Fulbert Nkwele Mangala, Cavin Epie Bekolo, Martin Ndinakie Yakum, Larissa Matcha Waffo, Bruno Kenfack

**Affiliations:** 1 Kekem District Hospital, Kekem, West Region, Cameroon; 2 Department of Biochemistry, University of Dschang, Dschang, West Region, Cameroon; 3 Faculty of Medicine and Pharmaceutical Sciences, University of Douala, Douala, Cameroon; 4 Maternity unit, Nkongsamba Regional Hospital, Nkongsamba, Littoral Region, Cameroon; 5 Department of Public Health, Faculty of Medicine and Pharmaceutical Sciences, University of Dschang, Dschang, West Region, Cameroon; 6 Department of Epidemiology and Biostatistics, School of Medical and Health Sciences, Kesmonds International University, Mile 3 Nkwen, Bamenda, Cameroon; 7 Department of Obstetrics / Gynaecology and Maternal Health, Faculty of Medicine and Pharmaceutical Sciences, University of Dschang, Dschang, West Region, Cameroon; PLOS: Public Library of Science, UNITED STATES

## Abstract

Short birth interval remains a major reproductive health problem, especially in the developing world. It is associated with maternal, neonatal, infant and under-five morbidity and mortality. This study identifies the risk factors of short birth interval among women in Cameroon. Women in early postpartum (with at least one previous live birth) from four health facilities in the Nkongsamba Health District were consecutively included in the study from September 2021 to December 2021. All data were collected by interview, using a semi-structured questionnaire, and analysed in Epi-Info version 7.2.3.1. With a statistically significant threshold of 5%, the adjusted odds ratio was used in multiple logistic regression to measure the association between short birth interval and potential factors. This study included a total of 679 participants with an age range of 18 to 47 years. Short birth interval was recorded in 46.10 [95%CI: 42.38–49.86]% of these women. A little more than half (56.72%) had at most 4 pregnancies already, while only 06.35% had at least 8 pregnancies in their reproductive life. Maternal age ≤ years (AOR = 2.66[1.80–3.93]), less than or equal to 10 months of breastfeeding of the previous child (OR = 2.48[1.80–3.41]), use of modern contraception before conception (AOR = 0.62 [0.43–0.89]), and the number of household occupants below 5 (AOR = 0.60[0.40–0.92]) were significantly associated with short birth interval. Short birth interval remains a significant call for concern in Cameroonian women. The likelihood of short birth interval is affected by maternal age, duration of breastfeeding, use of modern contraception and number of household occupants. Interventions to promote effective breastfeeding and postpartum family planning uptake are indispensable in the fight against short birth interval in Cameroon.

## Introduction

Adequately spacing births offers a chance for the woman to recover from the effects of the previous pregnancy. Short and long birth intervals have been described to be associated with a significantly higher risk for adverse maternal, perinatal and child outcomes. According to current World Health Organisation recommendations, the gap between the previous live birth and the subsequent delivery should be at least 33 months [1]. This implies a spacing of at least 24 months between the last live birth and the next pregnancy.

Short birth interval constitutes a major health problem, especially in low- and middle-income countries. According to the results of the 2018 Cameroon demographic and health survey, 50% of deliveries took place 31.2 months after the previous delivery. Up to 24% of deliveries occurred 24 months after the last pregnancy [2]. In sub-Saharan Africa, the prevalence of short birth interval has been reported in studies to remain consistently high, varying from 23% to 56% [3–7].

According to an analysis from an Ethiopian study, neonatal mortality is about 85% higher among women with short birth intervals compared to women without short birth intervals. The odds of infant mortality and under-five child mortality were doubled in women with short birth intervals compared to their counterparts without short birth intervals [8]. Similar findings of the adverse effect of short birth intervals on neonatal, infant and under-five mortality have been reported in other studies and systematic reviews [9–12]. It has been associated with preterm birth and low birth weight, which are major causes of neonatal mortality [10,11,13].

Multiple studies have been conducted in Africa to identify factors associated with short birth interval among women. These factors include duration of breastfeeding, socioeconomic level, level of education of the woman, use of contraception, parity, gender of the preceding child, religion (being a Muslim), age of the woman, age of the woman at her first delivery, number of living children, employment status of woman and husband, place of residence (urban/rural), and the level of education of the husband [1,3–5,14–22].

With the low level of modern contraceptive use and the high level of unmet need for family planning in Cameroon [23–26], the prevalence of short birth interval among Cameroonian women is likely to be high. Apart from reports of the national demographic and health survey, no studies have been carried out in Cameroon to present the prevalence of short birth interval in women of childbearing age. Studies to identify context-specific risk factors associated with short birth interval in Cameroon are inexistent. Identifying these factors is indispensable in the fight against short birth interval in Cameroon and its adverse effects on neonatal, infant and under-five mortality in Cameroon. This study was designed to determine the prevalence and risk factors of short birth interval among women in early postpartum of the Nkongsamba Health District, Cameroon.

## Materials and Methods

### Study design

We conducted a hospital-based cross-sectional study targeting women with at least one previous live birth who delivered in four health facilities of the Nkongsamba Health District. All data were collected through interview using a semi-structured questionnaire and analysed using the statistical software Epi-info version 7.2.3.1 [27].

### Setting and duration

Our study was conducted in Nkongsamba city, the capital city and major city of the Moungo Division, in the Littoral Region of Cameroon. Nkongsamba is located some 149.5 km from the economic capital of Cameroon, on the national highway number 5. It is characterised by a cosmopolitan population dominated by the Mbo’os. The Nkongsamba Health District constitutes one of the largest health districts of the Moungo Division. Based on patient turnover, four major health facilities including the Nkongsamba Regional Hospital, the Catholic Medicalised Health Centre, The Bon Samaritain Medicalised Heath Centre and the Fultang polyclinic. These health facilities receive and deliver 70–90% (depending on the year) of the pregnant women in the health district. Our study was conducted between September 2020 and December 2021.

### Study population and eligibility criteria

Our study population was made up of women of childbearing age from the Nkongsamba Health District who have a history of at least one previous live birth. We included all women with at least one previous live delivery who had just delivered in the maternities of the four health facilities chosen for this study.

### Sampling and sample size

This study’s minimum required sample size was estimated using the single proportion sample size formula. The expected prevalence of short birth interval was 56%, with the absolute precision on either side of the proportion set at 0.038 and an acceptable two-sided alpha error of 5%. This was used to estimate a minimum required sample size for this study of 656 participants. Participants were consecutively included as they delivered in the maternities of the four health facilities included until the required sample size was reached.

### Procedure of implementation

Administrative authorisations were obtained from the District Medical Officer of the Nkongsamba Health District and the Directors of the four health facilities included. When the protocol and data collection tools were ready, a pre-test of the questionnaire was conducted among 15 women of the Kekem District Hospital (West Region of Cameroon). After confirming the capacity of the questionnaire to meet research objectives while remaining participant friendly, we deposited the file to solicit an ethical clearance of the study. Data collectors who were holders of a degree in midwifery working in the respective maternities were trained in a working session which lasted for 5 hours. Key issues addressed were the steps to obtaining participant consent and how to collect the data required for the study.

### Data collection procedure

Data was collected using a semi-structured interviewer-administered questionnaire. This questionnaire collected data on the sociodemographic, economic, and obstetric characteristics of the participants. After presenting the study information, verbal consent from the participants was obtained. This was followed by a one-to-one interview of each participant during which the data was collected. Only willing and consenting participants were included in the study. The questionnaire evaluated the duration between the actual birth and the last live birth. It also collected data on sociodemographic characteristics (like age, level of education, religion, occupation, and the number of household occupants) and obstetric characteristics like the total number of pregnancies, abortion history, and stillbirth history.

### Definition of some study variable

Short birth interval: This study adopted the World Health Organisation’s definition of short birth interval, which considers at least 33 months between the previous live birth and the next delivery.

Use of modern contraception before conception: This included any woman who declared to have used a modern method of contraception between her last live birth and pregnancy.

### Data analysis

Data from questionnaires was keyed into a predesigned data entry sheet developed on Epi-Info version 7.2.3.1 and analysed. Selected quantitative variables were transformed into categorical variables. The prevalence of short birth interval was calculated as a proportion with its 95% confidence interval, while other characteristics of the population were presented as proportions. For normally distributed continuous variables, the mean age with its standard deviation was estimated. Potential risk factors of short birth interval reported in similar studies in other settings were selected and tested for their association with a short birth interval in our context. The strength of the association between short birth interval and the selected potential risk factors was measured using the odds ratio in simple logistic regression. All potential factors with p-values ≤0.25 were jointly included in a multiple logistic regression model, with one controlled for the effect of the others. The statistically significant threshold was set at 5%.

### Ethical consideration

This study was approved by the Cameroon Bioethics Initiative/Ethics Review and Consultancy Committee. For potential participants who could read and understand English, a copy of the information sheet was provided to them, and for those who could not read, the information sheet was read and explained to them. The information sheet contained the objective of the study, process of data collection, data confidentiality and expressly invited them to participate in the study while ensuring them of their liberty to accept or refuse participation and that their decision would not have any consequence on the healthcare they seek no or in the future. All participants were given enough time to ask questions and all their questions answered by the team. For this study, participants consent was verbal either given by them in case of an adult or by their parents or guidance in case of minors. This consenting process and the verbal consenting were approved by the ethics review board in the approved study protocol. Only participants who consented were included in this study.

## Results

### Characteristics of the study population

This study included a total of 679 participants with an age range of 18 to 47 years. The mean age of the participants was 30.32±5.68 years. Table 1 presents some sociodemographic and obstetric characteristics of the population. The most represented age group was the 21–30 years age group (52.25%), while less than 2% were aged between 15–20 years. A majority (65.43%) were in a union (cohabiting and legally married), while only 19.29% had acquired higher education. Most participants (42.69%) had between 100 and 200 thousand FCFA as monthly revenue, with households that mainly had 4–6 occupants (49.92%).

**Table 1:** Sociodemographic and obstetric characteristics of the study population.

Characteristic	Modalities	Frequency	Percentage
Age groups (n = 679)	15–20 years	13	01.91
	21–30 years	348	52.25
	31–50 years	318	46.83
Marital status (n = 674)	Cohabiting	296	43.92
	Single	231	34.27
	Married	145	21.51
	Widow	02	0.30
Level of education (n = 679)	Never schooled	02	0.29
	Primary	49	07.22
	Secondary	497	73.20
	Higher	131	19.29
Level of education of partner (n = 678)	Primary	16	02.36
	Secondary	483	71.24
	Higher	179	26.40
Religion (n = 668)	Atheist	43	06.44
	Catholic	322	48.20
	protestant	286	42.81
	Muslim	17	02.54
Occupation (n = 674)	Trader (Business)	85	12.61
	Teacher	73	10.83
	Housewife	273	40.50
	Student	73	10.83
	Others	170	25.22
History of stillbirth (n = 661)	Yes	78	11.80
	No	582	88.20
Duration of breastfeeding (n = 638)	< 7 months	140	21.94
	7–10 months	195	30.56
	>10 months	303	47.46
Use of modern contraception before pregnancy (n = 675)	Yes	334	49.48
	No	341	50.52
Estimated Monthly revenue in thousands of FCFA (n = 670)	Below 50	45	06.72
	Between 50 and 100	156	23.28
	Between 100 and 200	286	42.69
	Above 200	183	27.31
Number of household occupants (n = 661)	≤ 3	94	14.22
	4–6	330	49.92
	≥ 7	237	35.85
Total number of pregnancies (n = 677)	≤ 4	384	56.72
	5–7	250	36.93
	≥ 8	43	06.35
History of miscarriage (n = 664)	Yes	181	27.26
	No	483	72.74

A little more than half (56.72%) had at most 4 pregnancies already, and only 06.35% had at least 8 pregnancies in their reproductive life. History of at least one miscarriage was reported in 27.26% of the participants.

### Prevalence and risk factors of short birth interval

Short birth interval was recorded in 46.10 [95%CI: 42.38–49.86]% of these women. Table 2 shows the factors associated with short birth interval in our study population. A total of 14 potential factors were evaluated for their association with short birth interval. Following simple logistic regression, only four variables were found to have a statistically significant association with short birth interval. Women aged ≤30 years (OR = 2.49[1.82–3.40], p-value <0.001) and those who breastfed the previous baby for ≤ 10 months (OR = 2.48[1.80–3.41], p-value <0.001) had significantly higher odds of having a short birth interval compared to their respective counterparts. On the other hand, significantly lower odds of a short birth interval were observed when the number of household occupants was less than 5 (OR = 0.68[0.49–0.95], p-value = 0.025) or when the woman reported having used modern contraception before conception (OR = 0.49[0.36–0.67], p-value <0.001).

**Table 2:** Factors associated with short birth interval among the women.

Factor	Simple logistic regression		Multiple logistic regression	
	Odds Ratio [95%CI]	p-value	Adjusted Odds Ratio [95%CI]	p-value
Age ≤ 30 years (Y/N)	2.49 [1.82–3.40]	0.000*	2.66 [1.80–3.93]	0.000*
Duration of breastfeeding in last delivery ≤ 10 months (Y/N)	2.48 [1.80–3.41]	0.000*	2.18 [1.52–3.12]	0.000*
Level of education above secondary (Y/N)	0.95 [0.65–1.39]	0.787		
Level of education of partner above secondary (Y/N)	0.96 [0.68–1.35]	0.811		
Occupations other than teaching and business (Y/N)	1.11 [0.78–1.59]	0.568		
Number of living children below 4 (Y/N)	1.18 [0.85–1.64]	0.324		
Total number of pregnancies below 4 (Y/N)	1.21 [0.89–1.65]	0.212	0.98 [0.65–1.48]	0.918
Non-Catholic (Y/N)	1.21 [0.89–1.64]	0.216	1.23 [0.87–1.75]	0.234
Number of household occupants less than 5 (Y/N)	0.68 [0.49–0.95]	0.025*	0.60 [0.40–0.92]	0.019*
Use of modern contraception before pregnancy (Y/N)	0.49 [0.36–0.67]	0.000*	0.62 [0.43–0.89]	0.009*
Estimated monthly revenue below 50 thousand FCFA (Y/N)	0.78 [0.42–1.45]	0.430		
History of miscarriage (Y/N)	0.82 [0.58–1.15]	0.249	0.93 [0.61–1.42]	0.728
History of stillbirth (Y/N)	1.01 [0.63–1.62]	0.976		
Marital status Single (Y/N)	1.20 [0.83–1.73]	0.335		

Following multiple logistic regression in which seven variables with p-value ≤ 0.25 were controlled, the four abovementioned variables persisted with a statistically significant association with short birth interval. Women aged ≤ 30 years had a 2.66-fold higher odd for short birth interval compared to their older counterparts (AOR = 2.66[1.80–3.93], p-value <0.001). Also, women who breastfed their previous babies for ≤ 10 months had 2.18-fold higher odds for short birth interval compared to their counterparts who breastfed for a longer period.

On the other hand, women from households with less than 5 occupants had 0.60-fold lower odds for short birth interval compared to those from households with a higher number of occupants (AOR = 0.60[0.40–0.92], p-value = 0.019). Also, women who reported modern contraceptive use before pregnancy had 0.62-fold lower odds for short birth interval compared to women who did not (AOR = 0.62 [0.43–0.89], p-value = 0.009).

### Discussion

This study aimed to identify factors associated with short birth interval among women in the Nkongsamba Health District, Cameroon.

In this study, short birth interval was recorded in 46.10 [95%CI: 42.38–49.86]%. This implies that 4 to 5 women in a group of 10 in this population were at risk of having a short birth interval. The findings concord with the results of the 2018 demographic and health survey in Cameroon, which reported 50% of deliveries to have taken place 31.2 months after the previous delivery [2]. A systematic review in Ethiopia reported a pooled prevalence of 46.9%, similar to the findings of this study [5]. Exactly the same burden of short birth interval has been reported in a multilevel analysis of short birth interval in an Ethiopian survey [28]. In Uganda and Ghana, the prevalence of short birth interval was found to be 52% [4] and 49.7% [15], respectively.

On the other hand, some studies have found this prevalence to be significantly lower than our findings. Still, in Ethiopia (Northern Ethiopia), a prevalence of short birth interval as low as 23.3% has been recorded [3]. In Karachi, Pakistan, a prevalence of 22.9% was reported in 2021 [17]. In Nigeria, according to the analysis of the national demographic and health survey data, about 20% of women have a short birth interval. These significant discrepancies with our findings can be explained by the differences in contraceptive behaviours in the different settings. Contraceptive prevalence and behaviour depend on many sociodemographic and cultural factors [25,29–33], which vary from setting to setting. In addition, the average age at first pregnancy and delivery varies with settings and might affect the spacing couples want to leave between deliveries. This could also be affected by the desired number of children couples in different settings wish to attain.

In this study, younger age (≤ 30 years) was significantly associated with higher odds of short birth interval. Similar findings have been reported in Uganda, where younger maternal age was found to be associated with higher odds of short birth interval [4]. A similar association of younger maternal age with short birth interval has been reported in Bangladesh [16]. Moreover, women in Mbarara Hospital, Uganda, who were aged below 30 years had a 2.3-fold higher odd for short birth interval compared with older women [21]. Studies from Ghana [15] and Ethiopia [18] have also reported similar results. This could be explained by the desire of young women to end childbearing as fast as possible, have enough time to bring up the children and continue with their life activities.

Short duration of breastfeeding in the previous live birth was found to be associated with a short birth interval. This has been a consistent finding in multiple studies carried out in different settings [1,3,5,19,22,28,34]. Effective breastfeeding is a natural method of contraception. The more prolonged and more effective breastfeeding is carried out, the less likely the woman is to take in another pregnancy [35–37]. Even when breastfeeding can longer serve as a method of contraception, women with intentions of effective breastfeeding usually are engaged to take measures to avoid pregnancy. Encouraging women to breastfeed effectively for longer periods can serve as a means for the fight against short birth interval among women of childbearing age.

The use of modern contraception in the past before pregnancy was associated with lower odds of short birth interval. This association has been recorded in several other studies [1,5,17,38,39]. This brings to light the missed opportunity of postpartum family planning in preventing unintended pregnancy and short birth intervals. Encouraging women to use modern contraception between their pregnancies is indispensable in the fight against the adverse maternal and foetal outcomes associated with unintended pregnancy and short birth interval.

Women from households with less than 5 occupants had significantly lower odds of short birth interval compared to their counterparts with a larger number of occupants. In our search, we did not find this studies evaluating this association. Contrary to expectations that short birth intervals characterise households with fewer persons (small number of children), we found out that households with more than 4 persons had higher odds of short birth intervals. We feel that the number of household occupants might not be a predictor but an outcome associated with short birth interval. More studies need to be carried out on the relationship between the number of household occupants and short birth interval.

Contrary to findings reported in other settings, which state that level of education [17,34] and socioeconomic status [14,20] are predictors of a short birth interval, our results did not find any associations of short birth interval to these. It is likely that in Cameroon, these factors do not have a significant impact, or the sample size considered in our study did not have enough power to pick up these factors. However, context-specific variables might have affected these potential factors.

Cross-sectional studies fail to create cause-effect relationships and might even identify temporal associations. The factors presented in this study should therefore be interpreted carefully. However, our study was designed following adequate methodology with a control for confounding effects of other variables and presents a picture of the prevalence and risk factors of short birth interval in a sample of women in the Nkongsamba Health District, Cameroon.

#### Conclusions

The proportion of women with a short birth interval in the Nkongsamba Health District, Cameroon, remains unacceptably high. The likelihood of short birth interval among women significantly depends on maternal age, duration of the previous breastfeeding, use of contraception before conception and the number of household occupants. Encouraging postpartum family planning and effective breastfeeding will go a long way to reduce the prevalence of short birth interval and its consequences on maternal, neonatal and child health.

## Supporting information

S1 Database Data base on risk factors for short birth interval in Cameroon. (MDB)
